# Dynamics of snap-off and pore-filling events during two-phase fluid flow in permeable media

**DOI:** 10.1038/s41598-017-05204-4

**Published:** 2017-07-12

**Authors:** Kamaljit Singh, Hannah Menke, Matthew Andrew, Qingyang Lin, Christoph Rau, Martin J. Blunt, Branko Bijeljic

**Affiliations:** 1 Qatar Carbonates and Carbon Storage Research Centre, Department of Earth Science and Engineering, Imperial College London, SW7 2AZ London, UK; 2 Carl Zeiss X-ray Microscopy Inc., Pleasanton, CA USA; 3 Department of Earth Science and Engineering, Imperial College London, SW7 2AZ London, UK; 4 Diamond Light Source, Harwell Science and Innovation Campus, Didcot, UK

## Abstract

Understanding the pore-scale dynamics of two-phase fluid flow in permeable media is important in many processes such as water infiltration in soils, oil recovery, and geo-sequestration of CO_2_. The two most important processes that compete during the displacement of a non-wetting fluid by a wetting fluid are pore-filling or piston-like displacement and snap-off; this latter process can lead to trapping of the non-wetting phase. We present a three-dimensional dynamic visualization study using fast synchrotron X-ray micro-tomography to provide new insights into these processes by conducting a time-resolved pore-by-pore analysis of the local curvature and capillary pressure. We show that the time-scales of interface movement and brine layer swelling leading to snap-off are several minutes, orders of magnitude slower than observed for Haines jumps in drainage. The local capillary pressure increases rapidly after snap-off as the trapped phase finds a position that is a new local energy minimum. However, the pressure change is less dramatic than that observed during drainage. We also show that the brine-oil interface jumps from pore-to-pore during imbibition at an approximately constant local capillary pressure, with an event size of the order of an average pore size, again much smaller than the large bursts seen during drainage.

## Introduction

Two-phase fluid flow in permeable media is important in many processes such as water infiltration in soils, oil recovery from reservoir rocks, geo-sequestration of supercritical CO_2_ to address global warming, and subsurface non-aqueous phase liquid contaminant transport^1–5^. At the pore scale, capillary forces play a significant role in controlling fluid flow. During imbibition, in which the wetting phase displaces a non-wetting phase, the non-wetting phase can either exit the pore space completely during piston-like displacement, or it can become trapped (surrounded by the wetting phase). The trapping of the non-wetting phase is important: for CO_2_ storage, a maximum trapping efficiency is desired whereas for oil recovery, less trapping is preferable for efficient production. During water invasion in water-wet permeable media, the water in the corner layers in a throat – the restriction between two adjoining pore spaces – can swell until it is in a state of pressure non-equilibrium resulting in spontaneous filling of the throat and disconnection of the non-wetting phase^6, 7^. This process is called snap-off, which is a function of pore geometry and wettability^8^. Under a capillary-dominated flow regime and strong water-wetting conditions, snap-off is favorable for a large aspect ratio (the ratio of pore radius to throat radius)^8, 9^. The critical capillary pressure required for snap-off is always lower than that required for a piston-like displacement; however, snap-off can occur throughout the pore space, whereas piston-like advance is only possible from an adjacent pore that is already completely filled with the wetting phase^10^.

The pioneering two-dimensional micro-model experiments of Lenormand and co-workers^10, 11^, provided information on various pore-filling scenarios and the snap-off process. In three-dimensions, the dynamics have not been investigated in such detail; studies using X-ray micro-tomography, e.g., refs 12–16, report end states, i.e., after drainage and imbibition, which provide detailed information about ganglia or disconnected non-wetting phase clusters, but do not describe the dynamics of trapping. This time-dependent information is important to validate models of pore-scale displacement^17, 18^ and to quantify how the balance of viscous and capillary forces controls the exact nature of trapping.

With advances in synchrotron imaging, it is now possible to image real-time displacement processes in porous media^4, 5, 19–23^. Recently, synchrotron imaging has been used to examine drainage at the pore scale in sandstones and carbonate rocks^4, 5^ and desaturation of trapped oil clusters^21^. Rücker and co-workers^19^ studied imbibition in a sandstone sample, and reported snap-off and coalescence events that are linked to pore-scale viscous effects. They also reported cascades of multiple pore filling after a single snap-off or piston-like event. However, these studies have not quantified the capillary-pressure conditions under which pore-filling and snap-off processes occur during imbibition.

Here we present a study on the dynamics of displacement events during imbibition at reservoir pressure conditions. High-resolution fast synchrotron X-ray micro-tomography allows us to visualize and quantify the amount and rate of swelling of the brine layer leading to a snap-off process. The amount of swelling of the brine layer is then related to the local capillary pressure computed from the brine-oil curvature. We also compare the local capillary pressure before and after a snap-off event in drainage and imbibition processes. Furthermore, by analyzing pore-scale filling events from consecutive images during imbibition, we show that the brine-oil interface jumps from pore-to-pore with an event size of the order of an average pore size.

## Results and Discussion

The dynamic flow displacement experiments were conducted in a 3.8 mm diameter and 10 mm long Ketton limestone sample. The sample was first saturated with brine. The system was then pressurized to 10 MPa, followed by injection of oil (a drainage process for water-wet porous media) from the top of the sample by establishing a pressure difference of 50 kPa across ﻿both the rock sample and a low-permeability porous plate. The sample was imaged continuously during drainage using fast synchrotron X-ray micro-tomography with a voxel size of 3.28 µm and a time-step of 38 s between each image. When there was no longer any visible change in the oil and brine saturation, the flow was reversed by raising the pressure in the brine pump and establishing a pressure difference of 22 kPa to start brine injection (an imbibition process for a water-wet porous medium) from the base of the sample, achieving a capillary number (*N*_*C*_ = *vμ*/*γ*, where *v* is the Darcy velocity of the invading fluid, *μ* is the viscosity of the invading fluid, and *γ* is the brine-oil interfacial tension) of 1.26 × 10^−9^ representing a capillary-force dominated flow regime. Tomographic images were continuously recorded during imbibition.

From the analysis of the segmented data (binary image), we obtain a porosity (ratio of volume of pores to the total rock volume) of 0.120 ± 0.003 in the complete imaged sample. The initial oil saturation (ratio of volume of oil to volume of pores) after primary drainage (oil flooding) is found to be 0.558 ± 0.005. The final oil saturation (residual) after secondary imbibition (brine flooding) is 0.225 ± 0.003, resulting in a recovery of 59.6%. A complete three-dimensional image sequence of drainage and imbibition processes is shown in Movie S1 (Supplementary Information or https://figshare.com/s/bd4558d5ba52f32e2299 - DOI 10.6084/m9.figshare.4232330) and Movie S2 (Supplementary Information or https://figshare.com/s/55302a865e3fc0572c1d - DOI 10.6084/m9.figshare.4232354) respectively. The residual oil at the end of imbibition contains a number of disconnected oil ganglia (Movie S3, Supplementary Information or https://figshare.com/s/a6887930f5b73a40007d - DOI 10.6084/m9.figshare.4232324).

In the following sections, we explore the two most important pore-scale processes, i.e. pore-filling events (piston-like displacement) and snap-off resulting in the trapping of non-wetting phase (oil) during imbibition (brine flooding). Snap-off occurs when a piston-like displacement is not possible due to pore space accessibility. From the pore-filling (by brine) event analysis, we establish that the brine-oil interface jumps strictly from pore-to-pore which is controlled by the pore size. We start our discussion in the following section focusing on snap-off events (that result in oil trapping) for various pore topologies as well as fluid configurations, and show similarities in them. We then compare these snap-off events (in imbibition) with Roof-type snap-off events that occur during drainage (oil flooding).

### Snap-off of oil during imbibition

We observe snap-off events that lead to the trapping of 152 ganglia. Here, we analyze in detail three representative snap-off events with different local pore-space geometry and fluid configurations.

#### Snap-off during imbibition in a throat between two adjacent pores

**Figure 1:**
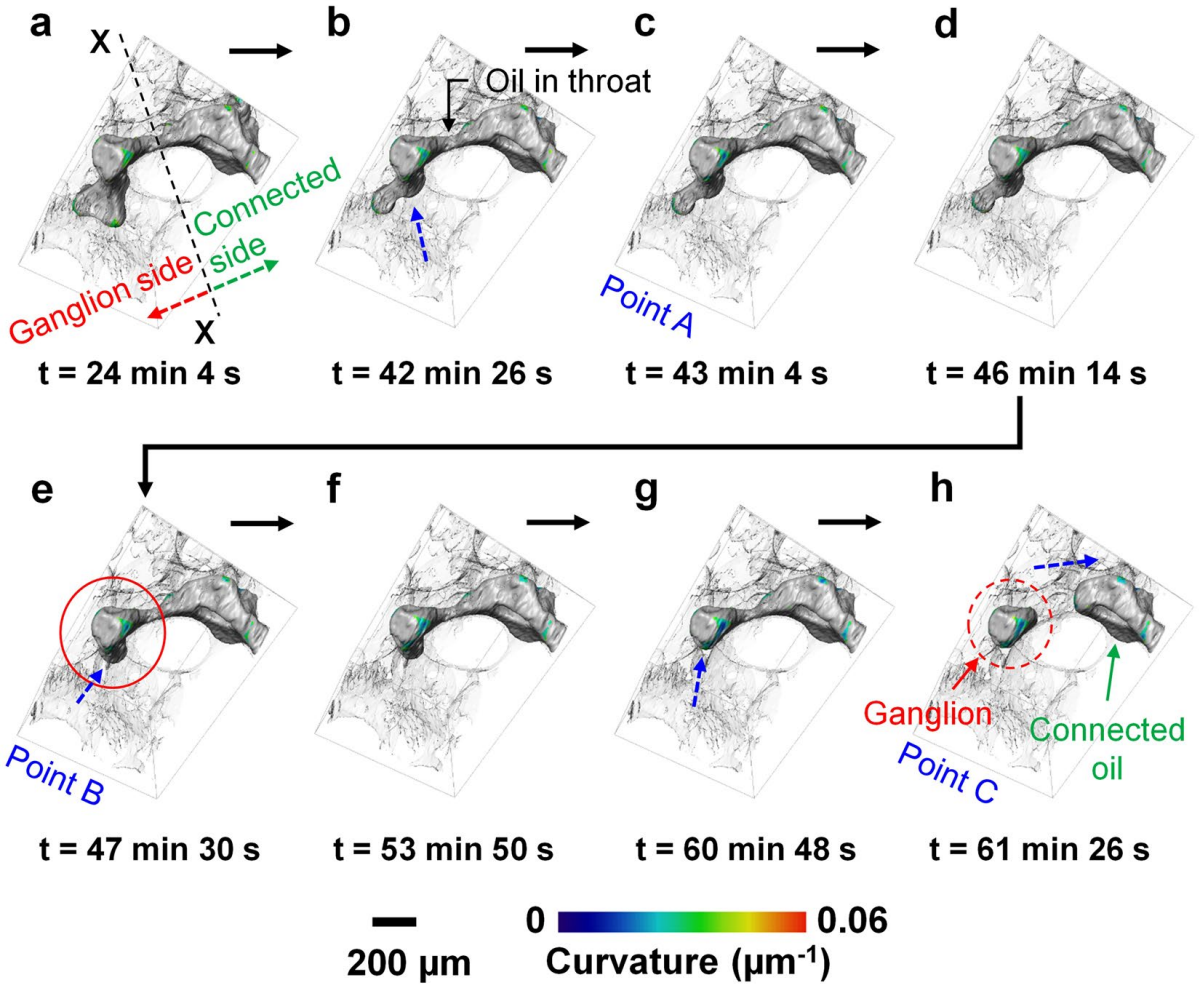
Snap-off during imbibition in a throat between two adjacent pores. Various times during brine injection showing oil progression and trapping in a water-wet Ketton rock sample: (**a**) t = 24 min 4 s (injected volume = 1.077 µL), (**b**) t = 42 min 26 s (injected volume = 1.899 µL), (**c**) t = 43 min 4 s (injected volume = 1.927 µL), (**d**) t = 46 min 14 s (injected volume = 2.069 µL), (**e**) t = 47 min 30 s (injected volume = 2.126 µL), (**f**) t = 53 min 50 s (injected volume = 2.409 µL, (**g**) t = 60 min 48 s (injected volume = 2.721 µL), and (**h**) t = 61 min 26 s (injected volume = 2.749 µL). The rock and brine are shown semi-transparent and transparent respectively for effective visualization. The dashed blue arrows indicate the approximate direction of interface migration. Points A–C show the events marked in Fig. 2. Time ‘t = 0’ denotes the start of the imbibition process (after acquiring the first tomographic image in imbibition). Time (t) represents the end of each tomographic image acquisition.

Figure 1a–h show snapshots during a pore-scale displacement and snap-off event that occurred in a throat between two adjacent throats resulting in trapping of the non-wetting phase in a single pore body during brine injection (imbibition). Two orthogonal grey-scale images of oil in the throat before snap-off occurred is shown in Figure S1 at t = 40 min 32 s. The complete three-dimensional sequence of the oil displacement process is shown in the Movie S4 (Supplementary Information or https://figshare.com/s/d0660fe226d90fb71e52 DOI - 10.6084/m9.figshare.4235381). During brine injection, the brine-oil interface on the ganglion-side (left of the X-X section – Fig. 1a) moves from pore to pore in a piston-like displacement without oil trapping (Fig. 1a–e). When the interface reaches the pore marked by the red circle (Fig. 1e), the brine occupying wetting layers in the adjacent oil-filled throat starts to swell. The brine layers continue to grow until the brine-oil interface is no longer stable in the throat, resulting in snap-off of the interface and trapping of the oil (formation of a ganglion). The swelling of the brine layer in the throat space is shown in Figure S2.

**Figure 2:**
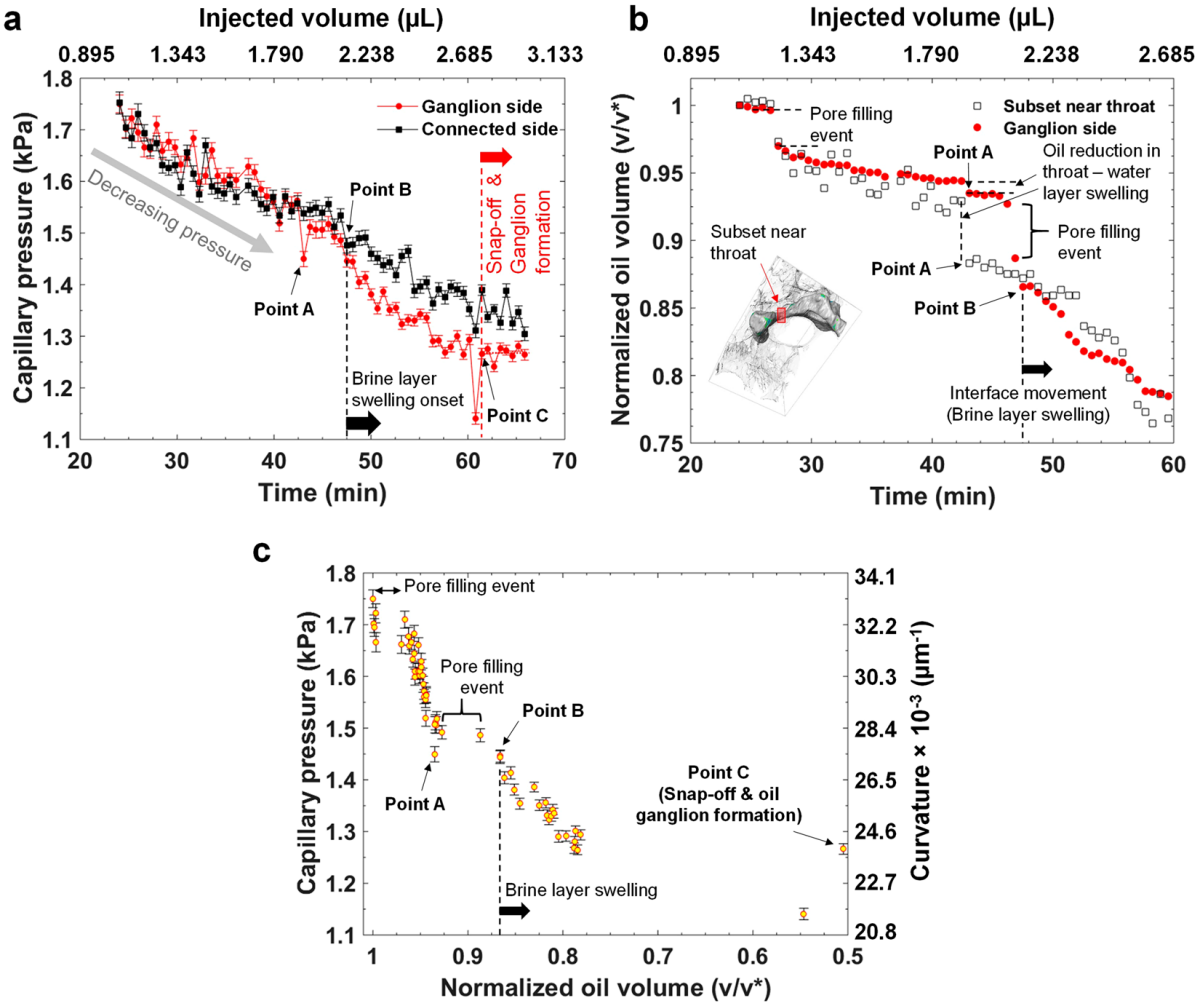
Local capillary pressure and oil saturation analysis during pore-filling and snap-off processes. (**a**) Capillary pressure, calculated from the total curvature using the Young-Laplace equation, is plotted against time and injected volume in the ganglion and connected side subsets that are shown in Fig. 1. (**b**) Oil volume (v) normalized to the oil volume (v*) at t = 24 min 4 s in the ganglion-side subset and in a subset near throat (marked by a red box in the inset picture) as a function of time and injected volume. (**c**) Capillary pressure of the brine-oil interfaces in the ganglion-side subset as a function of normalized oil volume (v/v*) in the ganglion-side subset. Here, the error bars in the capillary pressure are standard error in the mean with 95% confidence intervals calculated using ±1.96 × *σ*/√*N*, where *σ* is the standard deviation and *N* is number of points sampled.

To explain the snap-off process, we analyze the local capillary pressure (calculated from the the Young-Laplace equation, *P*_*c*_ = *γκ*, where *P*_*c*_ is the capillary pressure, *γ* is the interfacial tension between oil and brine, 52.33 ± 0.04 mN/m^24^, and *κ* is the total curvature estimated from an analysis of the segmented image) as a function of time and injected brine volume during the snap-off event observed in Fig. 1. First, we divide the oil surfaces into two regions, i.e. ganglion-side and connected side (left and right side of the X-X section respectively, Fig. 1a). The X-X section was selected in the throat where the snap-off occurred. Initially, oil in both subsets is connected to the bulk oil. With the injection of brine in the rock, the capillary pressure on both sides decreases approximately linearly with injected brine volume and time (Fig. 2a), and the brine-oil interfaces migrate from left to right. This is consistent with slow flow and interface movement governed by capillary equilibrium: this is thermodynamically a reversible process called an ison by Morrow^25^, see also ref. 26, and represents the piston-like filling of a pore as the wetting phase accesses larger regions of the pore space. At point A (Fig. 2a), a sudden decrease in capillary pressure corresponds to a rapid reduction in the oil volume in the subset near the throat (open symbols in Fig. 2b corresponding to the red box in the inset picture), demonstrating that the local capillary pressure gradient drives the brine flow towards the throat. Then just after 46 min, we see a pore-filling event between points A and B where the capillary pressure remains approximately constant (Fig. 2c) while locally the oil saturation decreases (Fig. 2b). This represents the rapid filling of a pore once the wetting phase has traversed the widest region of the pore space and reached the lowest, threshold, capillary pressure for filling. We see a very rapid change in local saturation, mediated by the flow of oil out of the center of the pore space. This type of advance, however, has not been seen or hypothesized hitherto: in drainage instead we see rapid changes in capillary pressure at a constant saturation – a rheon [Morrow^25^]. While there is some fluctuation in capillary pressure at the onset of the event, to within the uncertainty in the analysis, there is a largely constant capillary pressure during a rapid shift in oil volume.

Beyond point B, there is an apparent disparity in capillary pressure across the system: this represents a higher water pressure (lower capillary pressure) to the left, ganglion, side of the image in Fig. 1, driving water in layers to flow to the connected, right side. This represents a swelling of wetting layers, as can be seen by an incremental reduction in oil volume in the throat subset in Fig. 2b (open symbols). Eventually at point C, we see snap-off, and a disconnected ganglion of oil is formed. When this happens, there is a rapid change in capillary pressure and saturation with a change in the topology of the oil phase: this is a rheon event. The capillary pressure increases as the ganglion re-arranges in the pore space to minimize its surface energy at fixed volume.

The remarkable feature of the approach to snap-off event is that the swelling of wetting layers proceeds over approximately 14 minutes. This is many orders of magnitude slower than the sub-millisecond filling observed during a Haines jump in drainage^27, 28^, or indeed the change in saturation controlled by oil flow through the centers of the pore space seen here, which occur within the 38 s time-scale between scans. The reason for this difference is that the flow of water is slow – through wetting layers – whereas in drainage, the non-wetting phase advances through the centers of the pore space.

The flow rate calculated from the Poiseuille law, using an average capillary pressure drop between two sides (~72 Pa) of the throat with an approximate length of 600 µm and an equivalent radius of the amount of the brine (wetting phase) in the layers in the throat (at t = 0) ~28 µm, is ~29 nL/s, which is sufficiently fast to fill the throat in much less than 1 s. With this calculated flow rate, it is difficult to explain how the capillary pressure drop persists over a 10 min time-scale. Either there is brine flow outside the region studied, or the flow is much slower since it passes through sub-micron-scale intra-granular micro-porosity in the sample. There could be a systematic error in the curvature analysis and therefore in the estimation of the absolute values of capillary pressures: Figures S6 and S7 in the Supplementary Information indicate that the distributions of curvature on each point on the interface on either side of the throat overlap, with errors of order 10% dependent on the image segmentation. This warrants further investigation, both through imaging and numerical modeling, of flow and the associated local capillary pressure.

The capillary pressure required for snap-off is lower than the capillary pressure required for piston-like displacement without trapping, therefore, making piston-like displacement and pore-filling more favorable than a snap-off process for a pore of the same size, where possible. The capillary pressures for pore-filling and snap-off depend on pore geometry and the number of initially brine-filled adjacent throats. There has been extensive research in the past using two-dimensional glass micro-models for visualizing Roof-type snap-off in drainage, especially focusing on foam formation with applications to supercritical CO_2_ sequestration and enhanced oil recovery^29–31^, with some studies on snap-off in imbibition^8, 32^. This work suggests that the snap-off is dependent on the geometric and topological properties of the pore system^6, 8, 32^, as well as the initial location of the interfaces as explained by the analysis of pore-filling and snap-off rules by Lenormand and co-workers^10, 11, 33^. Our study confirms previously observed pore-scale mechanisms but provides the time-scales and mechanisms of brine layer swelling leading to oil snap-off in a three-dimensional porous medium.

#### Snap-off during imbibition at a pore junction – Case-I

**Figure 3:**
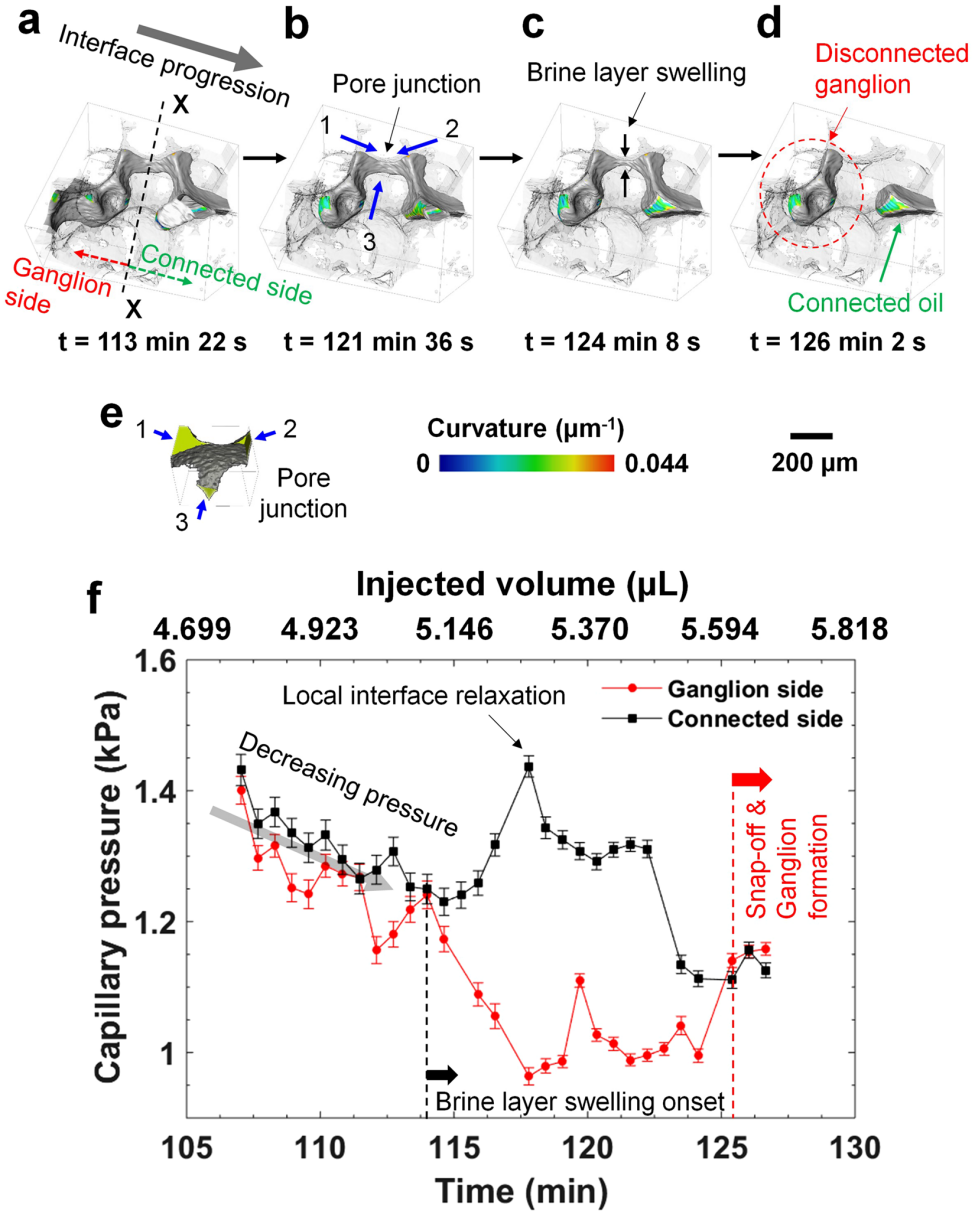
Snap-off during imbibition at a pore junction – Case-I. Various time steps during brine injection showing displacement of oil and trapping: (**a**) t = 113 min 22 s (injected volume = 5.073 µL), (**b**) t = 121 min 36 s (injected volume = 5.442 µL), (**c**) t = 124 min 8 s (injected volume = 5.555 µL), and (**d**) t = 126 min 2 s (injected volume = 5.640 µL). Time ‘t = 0’ denotes the start of the imbibition process (after acquiring the first tomographic image in imbibition). Each time (t) represents the end of each tomographic image acquisition. (**e**) Location of the pore junction where the snap-off occurred. (**f**) Capillary pressure in the ganglion and connected side is plotted against time and injected volume.

Figure 3a–d shows a sequence of pore-scale displacement of oil during imbibition, where snap-off occurred at a junction of three pores (marked by blue arrows in Fig. 3b and e), instead of a snap-off event in a single throat between two adjacent pores considered in the previous case. From the capillary pressure analysis on the ganglion-side and connected-side (Fig. 3f), we observe a similar behavior, namely a gradual capillary pressure drop over time representing the piston-like filling of a pore in local capillary equilibrium, followed by snap-off which is preceded by a period of over 10 minutes during which there is a significant capillary disequilibrium, driving the wetting phase to the throat through slow layer flow. The swelling of a brine layer is clearly visible in this example (Fig. 3c). Once the brine layer grows to a critical point (corresponding to a critical capillary threshold pressure), the oil in the throat can no longer remain stable and disconnects to create an isolated oil ganglion. The capillary pressure of the trapped ganglion, which is larger than the local threshold capillary pressure at the time of the snap-off event, remains constant with further brine injection.

#### Snap-off during imbibition at a pore junction – Case-II

**Figure 4:**
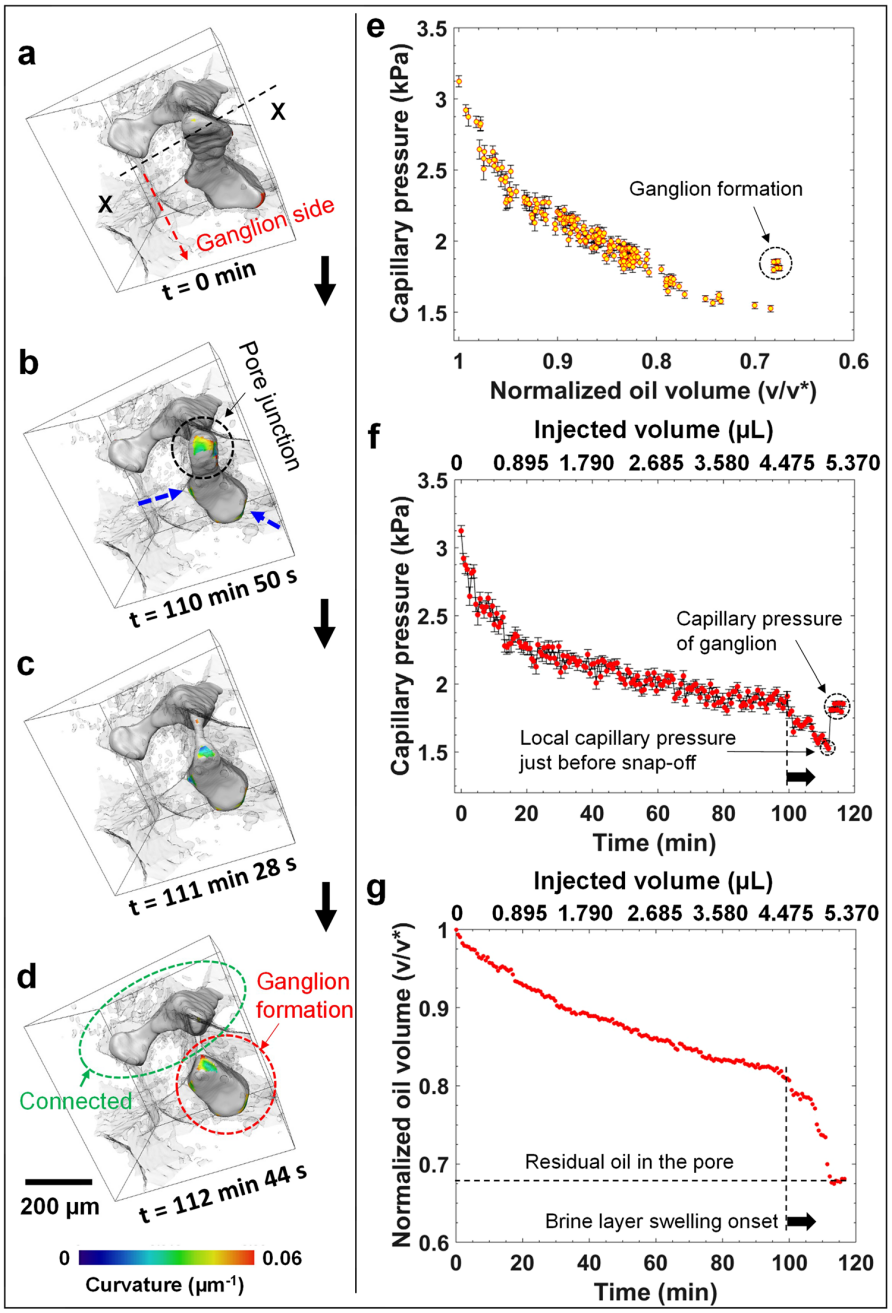
Snap-off during imbibition at a pore junction – Case-II. Various time steps during brine injection showing oil progression and trapping: (**a**) t = 0 min (injected volume = 0 µL), (**b**) t = 110 min 50 s (injected volume = 4.960 µL), (**c**) t = 111 min 28 s (injected volume = 4.988 µL), and (**d**) t = 112 min 44 ss (injected volume = 5.045 µL). The dashed blue arrows indicate the movement of the interface. (**e**) and (**f)** Show capillary pressure as a function of normalized oil volume and time respectively. (**g**) Normalized oil volume as a function of time.

We now present a third scenario in which snap-off also occurs at a junction of three pore spaces during imbibition. The difference in this case is that the brine-oil interface is already present in the same pore (established after drainage) where the ganglion formation is investigated. This is different from the first two cases, in which snap-off was proceeded by pore-filling events. Snap-shots of various time steps during ganglion trapping are shown in Fig. 4a–d. Figure 4e shows a plot of local capillary pressure on the ganglion-side as a function of normalized oil saturation. Here, the capillary pressure decreases smoothly with decreasing oil saturation without any pore-filling, as all the neighboring throats are already filled with brine. As the capillary pressure decreases with brine injection, the interfaces advance through pinning and de-pinning in a quasi-static motion (Movie S5, Supplementary Information or https://figshare.com/s/66c8d021e9e2229d6d27 - DOI 10.6084/m9.figshare.4235396). Once the critical capillary threshold pressure is reached, the interface in the throat junction is no longer stable and a snap-off event occurs, resulting in the trapping of oil ganglion in the pore body. Figure 4f shows a plot of capillary pressure as a function of time and injected brine volume, which is synchronized with the normalized oil saturation in the ganglion side subset as a function of time and injected brine volume (Fig. 4g). Similar to the previous snap-off events, we again observe a rapid decrease in the capillary pressure in the ganglion-side at the onset of brine layer swelling (marked by dashed vertical line). The oil saturation also decreases rapidly as the curved interface progresses towards the maximum pore opening. After snap-off there is a rapid rise in capillary pressure as the ganglion rearranges to find a lower energy configuration. The time-scale of the swelling of the brine layer leading to snap-off is ~12.7 min, which is similar to that observed in the previous cases investigated.

Overall, we observe similar mechanisms of brine layer swelling and snap-off irrespective of local pore-scale geometry and fluid configuration, as discussed in the previous sections. The time-scale for brine layer swelling in all the studied events are of the order of tens of minutes, followed by instantaneous snap-off processes. After snap-off, the local pore-scale capillary pressure rises as the ganglion rearranges to find a lower energy configuration.

### Pore-filling events and comparison of snap-off capillary pressure in drainage and imbibition

**Figure 5:**
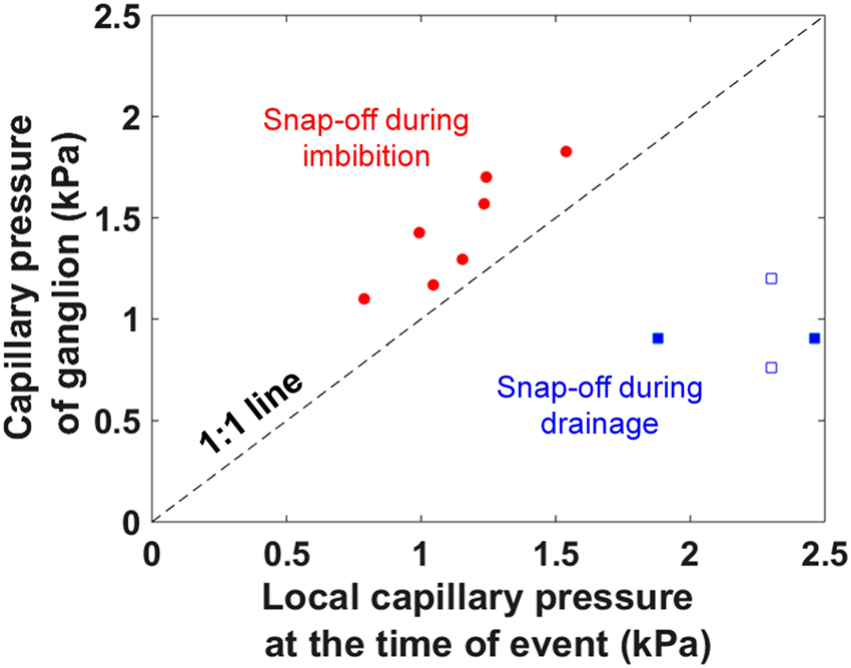
Comparison of local capillary pressures before and after snap-off events in drainage and imbibition. A plot of capillary pressure of trapped ganglia against the local threshold capillary pressure of the connected phase at the time of snap-off for both drainage and imbibition is shown. Data represented by filled symbols are from this work, and the data shown by empty symbols are for supercritical CO_2_-brine system (at 10 MPa and 50 °C) from Andrew *et al*.^4^.

A plot of the local threshold capillary pressure of the connected phases just before a snap-off event against the capillary pressure of trapped ganglia after the event, during both drainage and imbibition is shown in Fig. 5. There are two main observations from this plot. First, a ganglion formed during imbibition retains a higher capillary pressure than that of the local threshold capillary pressure just before a snap-off event. This is because the ganglion goes through rearrangements in the pore space after snap-off to minimize its free energy. As the ganglion pressure is higher, it is therefore not favorable to remobilize and reconnect the ganglion with the connected oil phase under the same flow conditions. However, in the case of a drainage event, a ganglion formed by Roof snap-off^34^ retains the capillary pressure that is lower than that of the local threshold capillary pressure just before the snap-off event: the reason again is that the trapped phase re-arranges itself in the pore space to minimize the oil pressure. This has also been observed in a recent study on drainage^4^. As the capillary pressure of the connected oil is larger than that of the disconnected phase, with further oil injection it can reconnect.

Secondly, the capillary pressure of the trapped ganglion is significantly different from that of the local threshold pressure just before the snap-off event for drainage compared to the imbibition process. This can be seen in Fig. 5, in which the data deviate far from the 1:1 line for drainage. In drainage, ingress through a single narrow throat can engender a cascade of filling in wider regions of the pore space. These Haines jumps can be of arbitrary size with a power-law distribution predicted from percolation theory, as observed in direct imaging experiments^5, 20^. After a Roof-type snap-off process, a trapped ganglion may re-arrange itself in a wide pore with a much larger radius of curvature and hence lower capillary pressure than that before the event – when the non-wetting phase entered the narrow throat.

**Figure 6:**
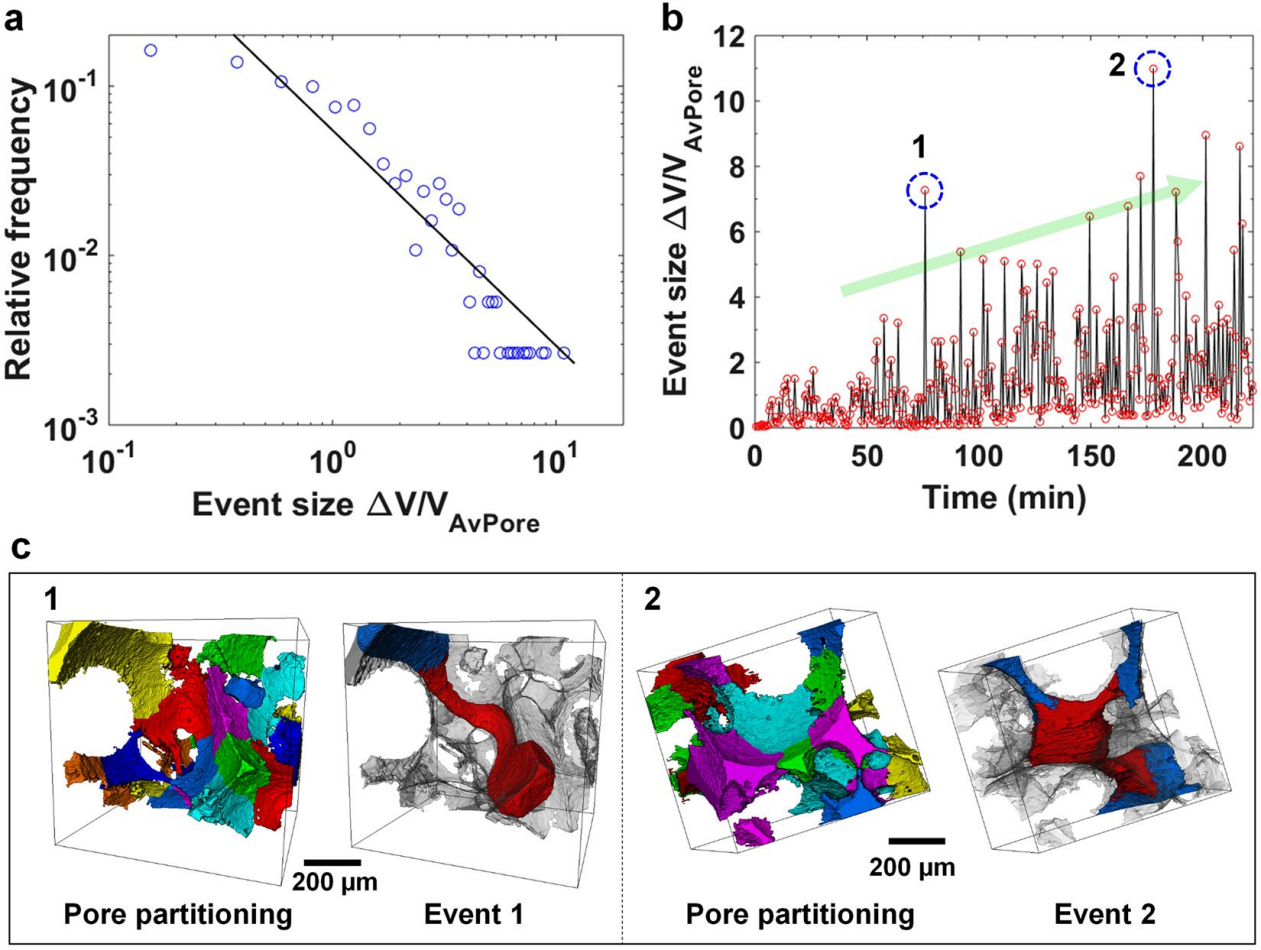
Pore-filling events. (**a**) A distribution of pore-filling (with brine) event volumes normalized by the average pore volume during imbibition. Here, only the largest event in consecutive tomographic images is presented. The line is a power-law fit, the exponent of which was calculated by a least absolute residual robust fitting algorithm. The smallest event size (the first data point) was excluded in data fitting. (**b**) The normalized size of pore-filling events as a function of time. (**c**) Pore-partitioned images showing subsets where event 1 and 2 (dashed blue circles in ‘Fig. 6b’) took place. Different colors in pore-partitioned images represent individual pores. In the event (1 & 2) images, red shows the locations where the pore emptied (oil was displaced by brine) after an event (obtained from subtracting consecutive images) and blue shows the new locations of the oil phase after an event. The complete pore space is rendered semi-transparent for effective visualization.

In an imbibition process, ingress is limited by the larger pore spaces; furthermore, wetting layer flow allows the invading fluid to access the entire system, allowing snap-off everywhere. This enforces a filling sequence that is more strictly related to size with more modest re-arrangements of trapped ganglia. This is shown in Fig. 6a in which a distribution of pore-filling (with brine) event volumes (normalized with average pore volume) is analyzed between consecutive tomographic images during imbibition. The procedure for obtaining the pore-filling events is explained in Figure S8 (also refer to the Material and Methods section). The distribution shows that the volume of a pore-filling event in imbibition is of the order of the average pore volume, which is about 2–3 orders of magnitude smaller than can occur in drainage^5, 20^. However, the distribution of event sizes follows an approximate power law with an exponent of −1.27, which is slightly lower than for a percolation-theory-type analysis where filling occurs in a disordered system in order of size^35^.

Figure 6b shows the normalized pore volume of a pore-filling event as a function of time. It appears that the volume of pore-filling events increases with time. This increase has also been observed in a recent study^19^, and can be linked to the decreasing capillary pressure. As the capillary pressure decreases, we can expect larger pores to be invaded by brine. Figure 6c (1–2) represents two subsets showing large volume pore-filling events, marked by dashed blue circles in Fig. 6b. Here, pore-filling events (event 1 & 2) are shown in dark red (by subtracting consecutive images), and individual pores in the neighborhood of a pore-filling event are shown in different colors. Although the volume of the events is larger than the average pore volume, the brine-oil interface jumps from a pore (after an event) and stabilizes in adjacent pores, therefore suggesting pore-to-pore filling. Cascading or interface jumps can only occur in smaller neighboring pores (as it appears in Event 1 in Fig. 6c-l in which an event occurs over an extended corridor of the pore space) or in throats until a new position of capillary equilibrium is reached.

## Conclusions

This study provides a pore-scale understanding of the dynamics of two-phase fluid flow during brine injection (imbibition) in a water-wet carbonate rock. We have used fast synchrotron X-ray micro-tomography to investigate fluid saturation, pore-scale brine-oil curvature, and pore-filling and snap-off events.

By analyzing the local capillary pressure calculated from brine-oil curvature data, we have investigated snap-off events with different local pore-space geometry and fluid configurations. The local capillary pressure on the ganglion side, where the oil becomes trapped, decreases at the onset of a snap-off event and creates a capillary pressure – and hence fluid pressure – gradient across the pore, driving the swelling of wetting layers until the threshold capillary pressure is reached and snap-off occurs. This process takes an order of 10 minutes, as opposed to the sub-second movement of fluid interfaces through the centers of the pore space previously observed in drainage. When a threshold pressure is reached, the brine-oil interface becomes unstable resulting in snap-off and trapping of the oil phase. After the snap-off event in imbibition, the oil re-arranges in the pore space to find a new position of minimum energy and the local capillary pressure rises, becoming higher than that in the connected non-wetting phase. On the other hand, a ganglion formed by Roof snap-off during drainage retains the capillary pressure that is lower than that of the local threshold capillary pressure just before the snap-off event: the reason again is that the trapped phase re-arranges itself in the pore space to minimize the oil pressure. We have also identified pore-filling events during imbibition in which the local capillary pressure remains approximately constant while locally the oil saturation decreases, representing the rapid filling of a pore space once the wetting phase has traversed the threshold capillary pressure for filling.

A comparison of the capillary pressure of ganglia and the local threshold capillary pressure of the connected phase just before a snap-off event reveals that the pressure fluctuates more dramatically in drainage compared to imbibition, with fluid movement usually limited to a single pore. This is because wetting layer flow during imbibition allows displacement throughout the pore space in an order more strictly dependent on size than in drainage. We also show that the brine-oil interfaces jump from pore-to-pore during imbibition with an average pore-filling events size of the order of an average pore size, which is much smaller than the large bursts observed during drainage. The pore-filling (by brine) event size analysis indicates that the volume of the largest event (between consecutive images) increases with time (or injected brine volume). This is linked to the decreasing capillary pressure, resulting in the invasion of larger pores.

## Materials and Methods

### Materials

The experiment was conducted on a 3.8 mm diameter and 10 mm long Ketton limestone rock sample from the Ketton quarry, Ruthland, UK, which contains >99% calcite with the remaining fraction being quartz^14, 36^. These samples were cleaned with methanol using Soxhlet extraction apparatus for 24 hours, followed by drying in a vacuum oven at 100 °C for 24 hours. A solution of potassium iodide (KI) salt (puriss, 99.5%, Sigma-Aldrich, U.K) in deionized water with a salinity of 1.8 M was used as the aqueous phase, which provided an effective X-ray contrast between brine and oil. Decane (ReagentPlus, ≥99%, Sigma-Aldrich, U.K.) was used as the oil phase. Decane was filtered four times through a column of aluminum oxide powder to remove surface active impurities and to obtain a stable brine-oil interfacial tension^37^.

### Sample preparation and experimental apparatus

The experiments were conducted in a Hassler-type flow cell made of carbon fiber that is nearly transparent to X-rays^14^. The cylindrical sample was placed on the top of a low-permeability water-wet porous plate (3.8 mm diameter and ~ 4 mm long) made of an Aluminum-silicate ceramic with a breakthrough pressure of 1.5 MPa (Weatherford Laboratories, Stavanger, Norway) in a Viton sleeve. The Viton sleeve was attached to a metal end piece at the base, which was connected to a syringe pump (ISCO-100D, TELEDYNE, U.S.A.) through PEEK tubing (Kinesis, U.K.). A PEEK spacer, with outer diameter of 4 mm and wall thickness of 1 mm, was placed at the top of the sample, which enabled monitoring of the brine-oil interface prior to drainage (oil injection). Before starting oil and brine flooding, the rock was imaged (hereafter called the dry reference scan).

The flow loop of the experimental apparatus is shown in Figure S3. After dry scanning (without any fluids) of the rock, it was flushed with CO_2_ to displace air followed by 80–100 pore volumes (PV) of brine injection at 0.1–0.2 mL/min to remove CO_2_ dissolved in brine, ensuring 100% brine saturation. The oil interface was carefully brought to the upper metal end piece that was attached to the oil pump through PEEK tubing. This was then carefully attached to the upper part of the Viton sleeve (on the top of the brine-filled PEEK spacer), avoiding air entrapment during assembly. The pressure in the brine, oil and the confining fluid (deionized water) was then raised step-wise to 10 MPa and 11.2 MPa respectively. A higher confining pressure was used to confine the Viton sleeve in which the sample was mounted to avoid any fluid bypassing along the walls of the rock sample. All the pumps were previously calibrated and tested for pressure difference by interconnecting and pressuring them. All the experiments were conducted at ambient temperature (20 °C).

### Oil and brine injection

For oil flooding (a drainage process in a water-wet porous medium), the pressure of the oil pump was raised by 50 kPa, which resulted in the migration of water-oil interface in the PEEK spacer. This interface was carefully monitored, and when the oil reached the top of the sample, continuous acquisition of the tomographic images was started. The low-permeability porous plate at the base of the core sample helped in controlling the movement of brine-oil interface before the entry point in the sample before drainage. It also provided low flow rates (due to its low permeability), which were important to acquire distortion free time-resolved tomographic images (discussed in the next section). This water-wet porous plate prevented the non-wetting phase (oil) passing through at the pressure drop used in this experiment, therefore, removing the dead volume during brine flooding as the oil-brine interface was established just at the base of the core sample after drainage. When there was no visible change in the oil and brine saturation (found by subtracting the projection data of consecutive tomographic images), the flow was reversed by raising the pressure in the brine pump and establishing a pressure difference of 22 kPa to start water injection (an imbibition process for a water-wet porous medium) from the base of the sample. A flow rate of 44.75 nL/min was achieved during imbibition, leading to a Darcy velocity of 3.94 µm/min and a capillary number (*N*_*C*_ = *vμ*/*γ*, where *v* is the Darcy velocity of the invading fluid, *μ* is the viscosity of the invading fluid, and *γ* is the brine-oil interfacial tension) of 1.26 × 10^−9^, using an interfacial tension of 52.33 ± 0.04 mN/m)^24^, representing a capillary-flow regime.

### Synchrotron imaging

The dynamic X-ray micro-tomography was performed at the Diamond Light Source (UK), on the Diamond-Manchester Imaging Branchline (I13-2) of Beamline I13, using a pink beam with photon energies up to 30 keV. The low energy X-rays were filtered by placing a set of 0.2 mm pyrolitic carbon, 2.2 mm aluminum, and 0.1 mm gold filters in the beam, which controlled the heating of the sample due to the absorption of low energy X-rays by the sample. The X-rays were converted to visible light by using a 250 µm thick CdWO4 scintillator; these photons were then recorded by a PCO Edge camera.

Tomographic images with a size of 2000^3^ voxels were acquired at a voxel size of 1.64 µm, which were then binned (2 × 2 × 2) to obtain images of 1000^3^ voxels with a voxel size of 3.28 µm. A total of 3000 projections with an exposure time of 0.06 s were acquired over 180° rotation for scanning the dry rock sample before starting the flow experiment. For dynamic imaging during drainage and imbibition processes, we collected 800 projections with an exposure time of 0.02 s for each tomographic image. Total acquisition time for each dynamic tomographic image was 24 s (16 s for acquisition and 8 s for triggering). The real time-step between each image was 38 s (which included 14 s for repositioning the rotation stage and transferring the data to a storage disk). We collected a total of 510 tomographic images during drainage and 434 images during imbibition.

### Image processing and analysis

The tomographic images were reconstructed using a filtered back-projection algorithm^38^. A cylindrical mask equivalent to the diameter of the rock sample was applied on the reconstructed data to remove unwanted regions including the Viton sleeve, followed by its conversion from 32-bit to 16-bit to reduce the size, using ImageJ software (http://imagej.nih.gov/ij). Hereafter, all the image-processing steps were performed using Avizo-9 software (https://www.fei.com/software/amira-avizo/).

#### Filtering and oil segmentation

The images were filtered with a non-local means edge preserving filter^39, 40^. First, the filtered dry reference image (Figure S4-a) was segmented into two phases (pore and solid) with a seeded watershed algorithm based on the gray-scale gradient and gray-scale intensity of each voxel (Figure S4-b). All the dynamic time-series filtered images were then registered to the filtered dry reference scan using normalized mutual information and resampled onto the same voxel grid as the dry reference scan. Each time-series filtered tomographic image containing three phases (Figure S4-c) was then subtracted from the first brine-saturated image. The image subtraction not only helped in enhancing the contrast between oil and the other phases, but also canceled out the effect of phase-contrast (dark spots in the images). These subtracted images were then again filtered using a non-local means filter to increase the signal-to-noise ratio (Figure S4-d). These images were then segmented for the oil phase using intensity-based thresholding (Figure S4-e). Figure S5 shows a pore-scale inspection of the quality of non-local means filtering and oil segmentation. By comparing Figure S5a and b, it is clear that the filtering improves the signal to noise ratio significantly while keeping the phase boundaries preserved. Figure S5c shows that the segmentation process captured the oil phase boundary effectively. The oil-segmented data were used for curvature and capillary pressure analysis, discussed in the next section.

#### Brine-oil curvature mapping and capillary pressure

The brine-oil curvatures were obtained by creating best-fit quadratic surfaces at each point on the smoothed surface generated from the segmented data of Figure S4-e ^12, 36, 41^. First, the overall curvature distribution of the oil phase was obtained, and then the oil-brine curvature was extracted by isolating the boundaries of the brine-oil interface. This is described in detail in Singh *et al*.^12^; see their Fig. 4. Due to the sensitivity of segmentation near the three-phase contact line, which could affect brine-oil curvatures, the rock phase was dilated and applied as a mask on the oil curvature maps to remove the curvature values near the three-phase contact line. We examined the effect of the amount of rock dilation (number of voxels) on curvature distributions and their mean values along with their standard deviations. Figure S6(a–h) show an example oil surface from Fig. 1 (different orientation) at t = 25 min 58 s with 1–8 voxel dilation (of the rock phase) respectively. As the number of voxel dilation increases, the brine-oil curvature points near the three-phase contact line are removed, however, at the expense of the counts of curvature values, with almost no brine-oil curvature surface left behind at a 8 voxel dilation. Here, the idea is to obtain an optimal value that provides curvature values away from the rock surface, while keeping sufficient number of curvature values for statistics. Figure S6i provides the distribution of the curvature values obtained after voxel dilation presented in Figure S6(a–h). With an increasing number of voxel dilations, the distribution moves towards a Gaussian distribution for a 4–5 voxel dilation. With a further increase in the number of dilated voxels, we have a limited count to obtain a distribution. Figure S6(k–l) show the mean of the curvature values and standard deviation for these distributions. Except for the first data point for 1 voxel dilation, the mean value and the standard deviation decrease with the increasing numbers of voxel dilation. Based on the qualitative and quantitative observations from Figure S6, we selected 4 voxel rock dilation. The idea was to remove distorted curvature values near the grain boundaries in pores and throats, while keeping many curvature values for statistics. Figure S6j shows the distribution with 4 voxel dilation and a fitted Gaussian curve. One could argue between 4 and 5 voxel dilation based on these results. However, our selection is based not only on this distribution, but also for the curvature values presented in Figs 3 and 4, especially for Fig. 4 in which the count was significantly reduced at 5 voxel dilation.

Capillary pressure (*P*_*c*_) was calculated from the total curvature using the Young-Laplace equation.

1 $${P}_{c}={P}_{o}-{P}_{w}=\gamma \kappa $$

where *P*_*o*_ and *P*_*w*_ are the pressures in the oil and water phase respectively, *γ* is the oil-brine interfacial tension, and *κ* is the total curvature of the oil-brine interface. The mean value of the capillary pressure obtained from the distribution shown in Figure S6j is 1.68 kPa. Although the distribution shows a large spread with a standard deviation of 0.32 kPa, the data fit well with a smooth Gaussian curve. The standard error in the mean of the distribution with 95% confidence interval, calculated using ±1.96 × σ/√*N*, where *σ* is the standard deviation and *N* is number of points sampled, is found to be 0.01 kPa. This represents the uncertainty in the mean value of the capillary pressure for a Gaussian distribution.

A comparison of transducer based capillary pressure measurements to that measured from the brine-oil interfacial curvature is provided by Armstrong *et al*.^41^. They report a good agreement between these two measurements for imbibition, however, drainage shows a large error. The reason for this disagreement in the case of drainage could be that the brine-oil interfaces are in the close vicinity of a throat where the interfacial voxel count is limited, resulting in large errors in the curvature measurement. However, for imbibition, the results are in good agreement, the interfaces are in the wider regions of the pore space, therefore, giving a large voxel count and less error. This gives confidence to our analysis presented in this study on imbibition.

#### Sensitivity analysis of the local curvature on either side of a snapping-off throat

Figure S7 provides a comparison of the distribution of the mean curvature on either side of a snapping-off throat shown in Fig. 3 at different time steps during brine flooding (imbibition). For this analysis, we selected a time step (t = 111 min 28 s) where both mean values of the brine-oil curvatures (consequently capillary pressure) are similar (also see Fig. 3f) shown in Figure S7a, and two other time steps where the mean values of the brine-oil curvatures are significantly different, such as t = 118 min 26 s (Figure S7b) and t = 119 min 4 s (Figure S7c). In Figure S7a, the curvature distribution on the ganglion side and the connected side (either side of a snapping-off throat) overlap, with mean value of (12.11 ± 0.19) × 10^−3^ µm^−1^ and (12.09 ± 0.22) × 10^−3^ µm^−1^ respectively. However, the distributions are significantly different for Figure S7b (with mean values of (9.36 ± 0.1) × 10^−3^ µm^−1^ and (12.83 ± 0.16) × 10^−3^ µm^−1^ on the ganglion and connected side respectively) and Figure S7c (with mean values of (9.44 ± 0.09) × 10^−3^ µm^−1^ and (12.66 ± 0.14) × 10^−3^ µm^−1^ on the ganglion and connected side respectively). The difference in the curvature distributions on either side of the snapping-off throat are statistically analyzed using the Welch’s t-test for unequal variances^42^. In this analysis, the statistic ‘t’ is obtained from the mean values and standard deviation:

2 $$t=\frac{{\bar{x}}_{1}-{\bar{x}}_{2}}{\sqrt{\frac{{\sigma }_{1}^{2}}{{n}_{1}}+\frac{{\sigma }_{2}^{2}}{{n}_{2}}}}$$

where ${\bar{x}}_{1}$ and ${\bar{x}}_{2}$ are the mean values of curvature of two distributions on either side of a snapping-off throat (i.e., ganglion and connected sides), and *σ*_1_ and *σ*_2_ are the standard deviations, and *n*_1_ and *n*_2_ are the sample size for two distributions respectively. The degree of freedom (*f*) is calculated using the following equation:

3 $$f=\frac{{(\frac{{\sigma }_{1}^{2}}{{n}_{1}}+\frac{{\sigma }_{2}^{2}}{{n}_{2}})}^{2}}{\frac{{\sigma }_{1}^{4}}{{n}_{1}^{2}({n}_{1}-1)}+\frac{{\sigma }_{2}^{4}}{{n}_{2}^{2}({n}_{2}-1)}}$$

Comparing the value of calculated statistic ‘*t*’ obtained from equation (2) with the threshold ‘*t*’ obtained from ‘*t*’ table using equation (3) suggests that the two distributions in both Figure S7b and c are different from each other with more than 98% probability. On the other hand, the probability that the two distributions in Figure S7a are different is close to zero. This provide us confidence that the analysis and the interpretation of the local capillary pressure (obtained from the curvature distribution) on the either side of a snapping-off throat (ganglion and connected sides) used in this study are correct.

#### Pore-filling event analysis

The oil-segmented data were further analyzed for pore-filling event analysis during imbibition. The pore-filling (by brine) events were determined by subtracting the oil phase in two consecutive images. This is explained in Figure S8. Figure S8a shows a subset containing oil in blue with semi-transparent rock at t = 75 min 22 s. During imbibition, the oil was invaded by brine in the next time step at t = 76 min, marked by a dashed-red arrow in Figure S8b. The oil voxels between these time steps were subtracted to obtain the size of a brine-filling event. This is shown in Figure S8c in red. The brine-filling time scales obtained from this analysis, which is of 38 s, provides an upper bound on the actual time scale of a pore-filling event in which a brine-oil interface jumps between adjacent pores instantaneously (of the order of milliseconds). From each subtracted image, the volume of the largest subtracted oil cluster was selected for the analysis of the largest pore-filling events occurring between consecutive images. For the qualitative analysis of the pore space filled during these events, the pore space was isolated from the segmented data (Figure S4-b), and then separated into individual pores by computing watershed basins on a Euclidian distance map: these are wide regions of the void space bounded by throats. The throats represent the restrictions between two pores, with the smallest distance to the surface. A complete workflow of image processing for pore partitioning is described in Singh *et al*.^12^. The volume of each pore was calculated using an approach described by Dong and Blunt^43^.

#### Three-phase segmentation

For three-phase segmentation that was used for calculating phase volume fractions in the complete imaged rock, the dry reference segmented image from Figure S4-b was applied as a mask on oil segmented data (Figure S4-e). Figure S4-f shows the three-phase (brine-oil-rock) segmented data. Note that the masking resulted in an apparent brine layer near some of the grain surfaces (compare Figure S4-c and f). To correct this artefact, we adopted a sequential dilation and erosion routine where the oil phase was isolated and dilated by three voxels. Then the original grain segmented image (from the dry scan) was applied as a mask on it. The resultant brine phase was isolated and dilated by three voxels (eroding the dilated oil phase). The original grain segmented image was again applied as a mask to obtain the final image (Figure S4-g), resulting in the removal of the apparent brine layer while preserving the oil-brine interface. Figure S5d shows the quality of the three-phase segmentation at the pore scale.

## Electronic supplementary material

SUPPLEMENTARY INFORMATION

Three-dimensional image sequence of drainage in the complete imaged rock

Three-dimensional image sequence of imbibition in the complete imaged rock

Residual oil at the end of imbibition containing a number of disconnected oil ganglia

Snap-off during imbibition in a throat between two pores

Snap-off during imbibition at a pore junction
