# Supplementary material for: Dynamics of snap-off and pore-filling events during two-phase fluid flow in permeable media
Source: Sci Rep. 2017 Jul 12;7:5192. doi: 10.1038/s41598-017-05204-4 (PMC5507864; doi:10.1038/s41598-017-05204-4)
Supplement: Supplementary file 1 — SUPPLEMENTARY INFORMATION [file 41598_2017_5204_MOESM1_ESM.pdf]

SUPPLEMENTARY INFORMATION

Dynamics of snap-off and pore-filling events during two-phase fluid flow in permeable media

Kamaljit Singh[1]*, Hannah Menke[1], Matthew Andrew[1,2], Qingyang Lin[3], Christoph Rau[4], Martin J. Blunt[1], and Branko Bijeljic[1]

[1] Qatar Carbonates and Carbon Storage Research Centre, Department of Earth Science and Engineering, Imperial College London, SW7 2AZ London, U.K.

[2] Carl Zeiss X-ray Microscopy Inc., Pleasanton, CA, U.S.A.

[3] Department of Earth Science and Engineering, Imperial College London, SW7 2AZ London, U.K.

[4] Diamond Light Source, Harwell Science and Innovation Campus, Didcot, U.K.

* Corresponding author. Email address: kamaljit.singh@imperial.ac.uk

MOVIE & FIGURE LEGENDS

Movie S1. Three-dimensional image sequence of drainage in the complete imaged rock. Also available at https://figshare.com/s/bd4558d5ba52f32e2299 - DOI 10.6084/m9.figshare.4232330.

Movie S2. Three-dimensional image sequence of imbibition in the complete imaged rock. Also available at https://figshare.com/s/55302a865e3fc0572c1d - DOI 10.6084/m9.figshare.4232354.

Movie S3. Residual oil at the end of imbibition containing a number of disconnected oil ganglia. Also available at https://figshare.com/s/a6887930f5b73a40007d - DOI 10.6084/m9.figshare.4232324.

Movie S4. Snap-off during imbibition in a throat between two pores. Also available at https://figshare.com/s/d0660fe226d90fb71e52 DOI - 10.6084/m9.figshare.4235381.

Movie S5. Snap-off during imbibition at a pore junction. Also available at https://figshare.com/s/66c8d021e9e2229d6d27 - DOI 10.6084/m9.figshare.4235396.

Figure S1. Oil in pores and throats.

Figure S2. Brine layer swelling in the throat.

Figure S3. Experimental apparatus.

Figure S4. Image processing and segmentation.

Figure S5. Inspection of the quality of image processing.

Figure S6. Effect of rock dilation on curvature analysis.

Figure S7. Comparison of the distribution of the mean curvature of brine-oil interfaces on either side of a snapping-off throat.

Figure S8. Pore-filling events.

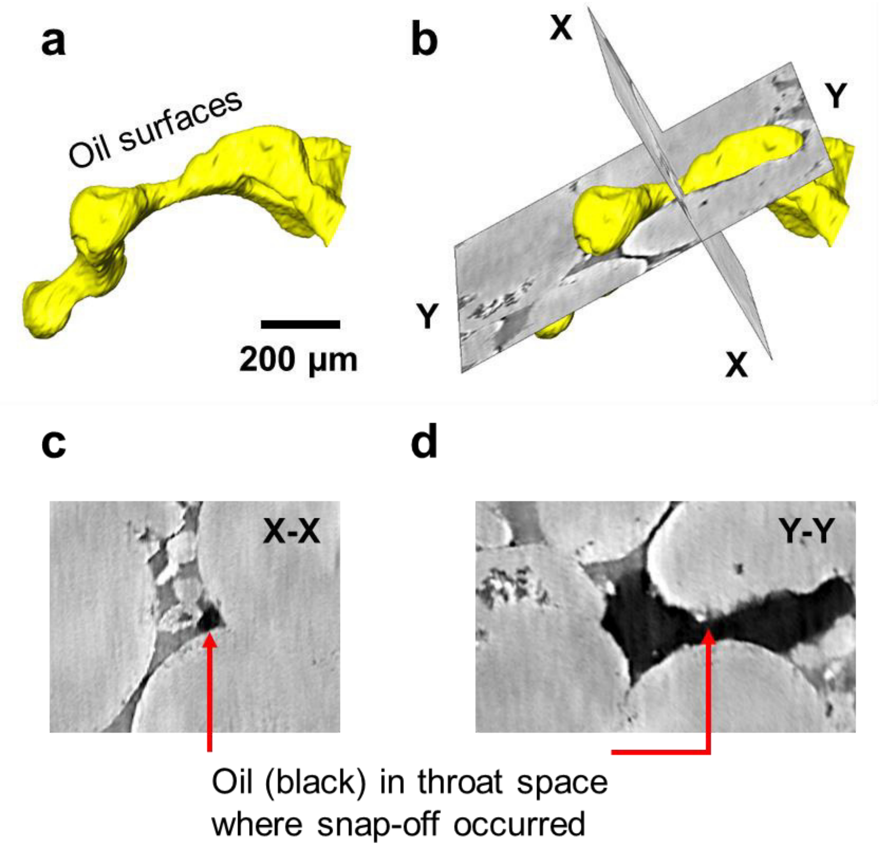

Figure S1. Oil in pores and throats. (a) Oil surface (yellow) at t = 40 min 32 s during imbibition. (b) Two-dimensional orthogonal grey scale images providing cross-sections of throat where snap-off occurred. (c-d) Re-oriented orthogonal images showing oil (black) in the narrow region (throat) where snap-off occurred. It is clear that the non-wetting phase stays connected in the throat space.

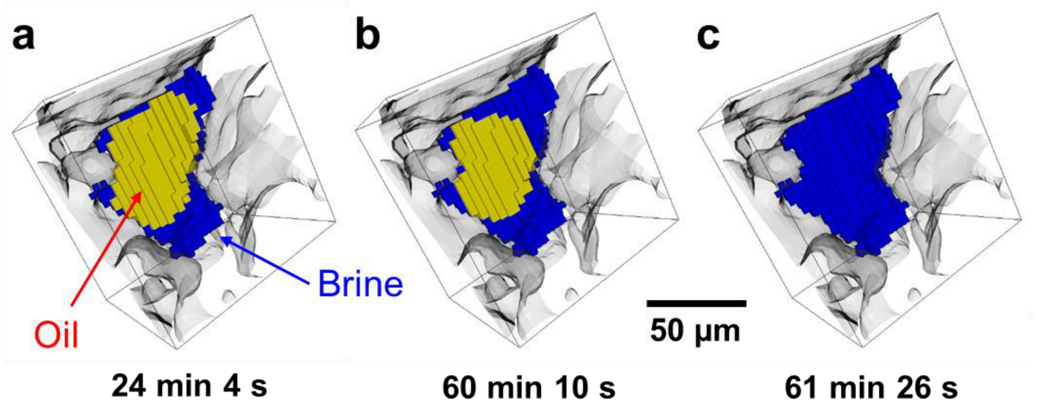

Figure S2. Brine layer swelling in the throat. Various time steps during brine injection showing the swelling of brine (blue) layers in the throat: (a) t = 24 min 4 s, (b) t = 60 min 10 s, and (c) t = 61 min 26 s (after snap-off). Oil is shown in yellow and rock semi-transparent. Here, only the voxels at the throat surface (that was obtained from pore-throat portioning using watershed basins on a Euclidian distance map) are shown.

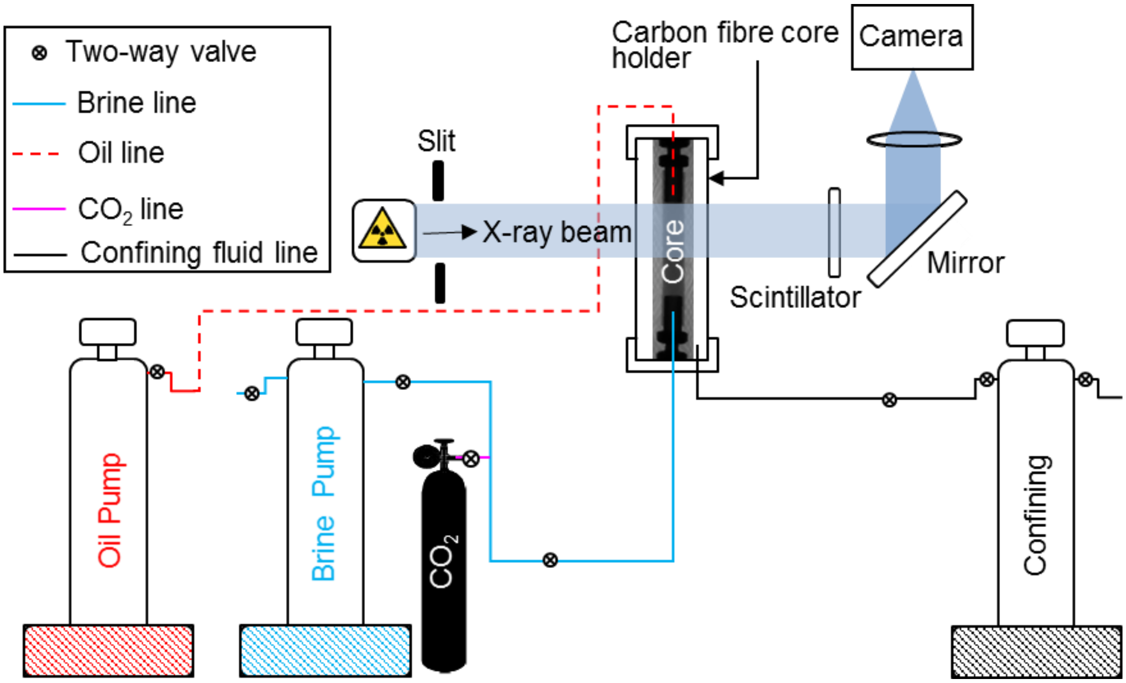

Figure S3. Experimental apparatus. The oil and brine pumps controlled the pressure of the fluids, while the confining pump maintained a pressure of 11.2 MPa confining the Viton sleeve in which the sample was mounted.

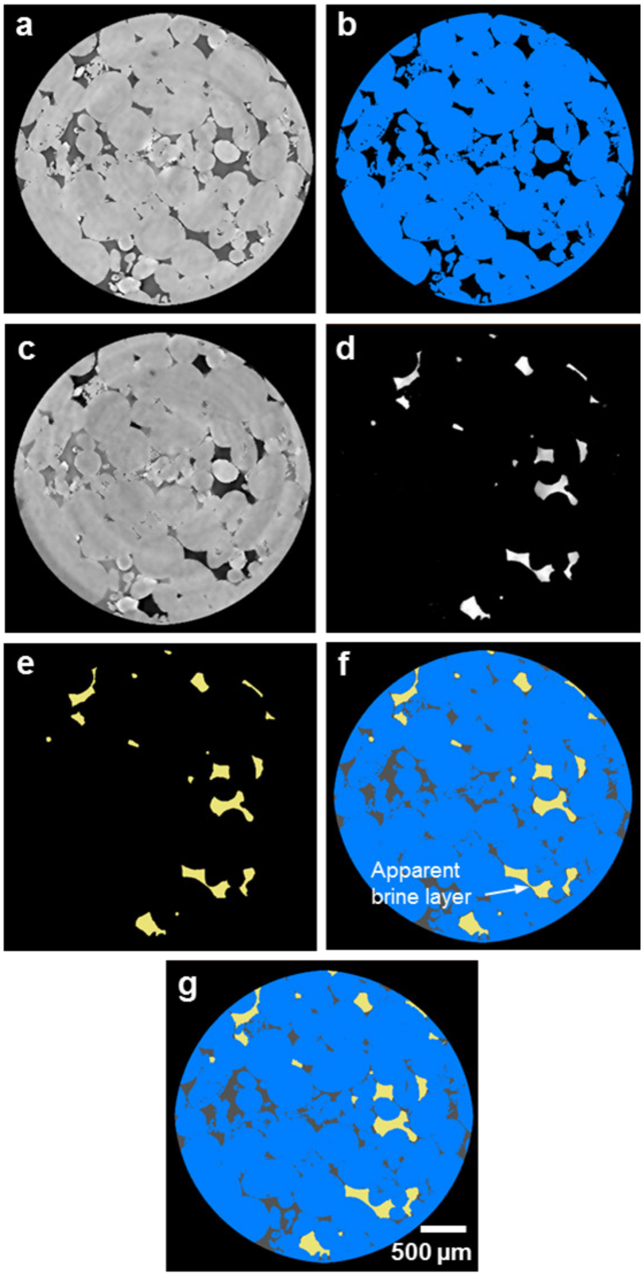

Figure S4. Image processing and segmentation. (a) Two-dimensional horizontal cross-section of the filtered dry reference scan. Here, light gray and dark gray represent rock and pore space respectively. (b) The image was segmented into two phases using a watershed algorithm. Here blue and black represent rock and pore respectively. (c) Two-dimensional horizontal cross-section of a three-phase filtered tomographic image. (d) The image was subtracted from the initial brine-saturated reference image and filtered with a non-local means filter. (e) The data were segmented for oil phase using an intensity-based thresholding method. (f) The grain data from dry reference image were applied as a mask to obtain the three-phase segmented image. Note that this sometimes created an apparent brine layer at the boundary of the grains. (g) The final corrected image, after applying a sequential dilation and erosion routine.

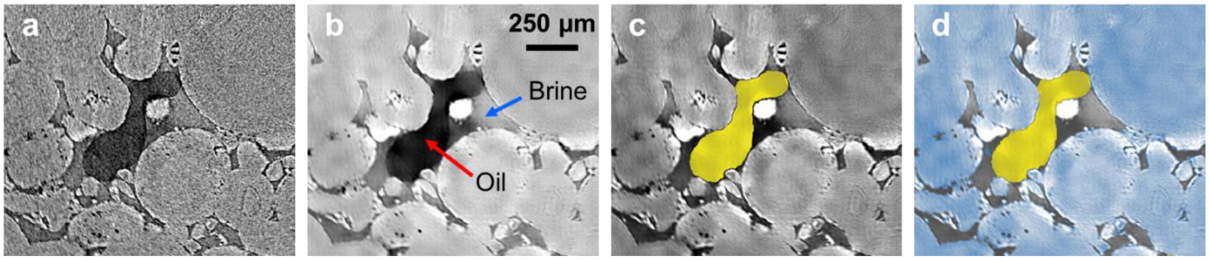

Figure S5. Inspection of the quality of image processing. (a) A two-dimensional cross-section of an original raw image showing various phases at t = 40 min 32 s. The same image after the application of a non-local means filter is shown in (b). (c) The oil segmented data is overlain on the filtered image to show the quality of segmentation. (d) Three-phase segmented image overlain on the filtered image, again showing the quality of three-phase segmentation using erosion-dilation method.

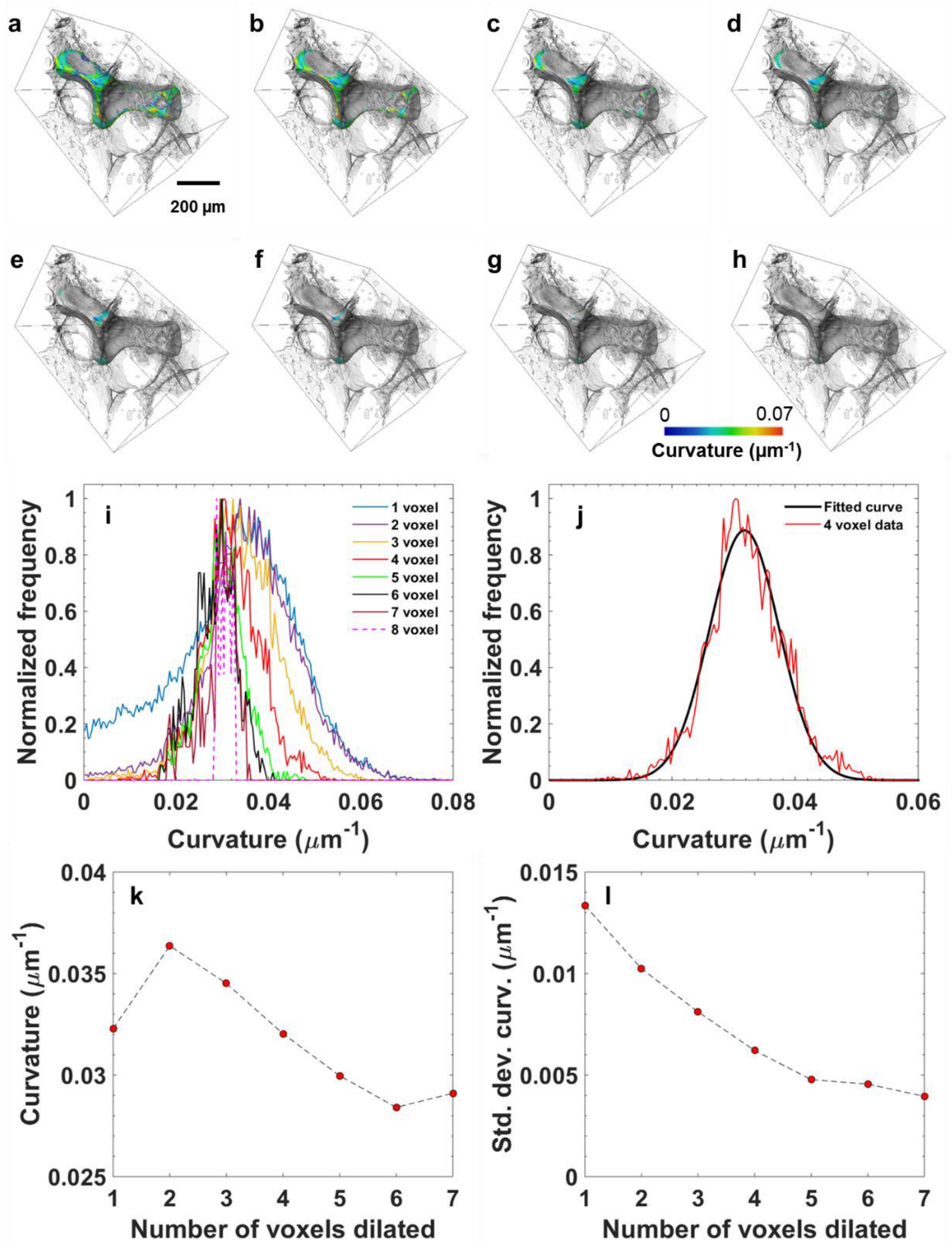

Figure S6. Effect of rock dilation on curvature analysis. (a-h) Curvature maps after 1-8 voxel dilation respectively. With the increasing amount of dilation, the curvature values near the three-phase contact points are removed at the expense of counts of the values across the surfaces. (i) Distribution of curvatures shown in (a-h). (j) Curvature distribution with 4 voxel rock dilation which was used in the manuscript. (k-l) Mean values and standard deviation of the curvature distributions as a function of number of voxel dilation. Here, the values for 8 voxel dilation are not shown as the count of curvature on surfaces is limited therefore significantly affecting the distribution as shown in (i).

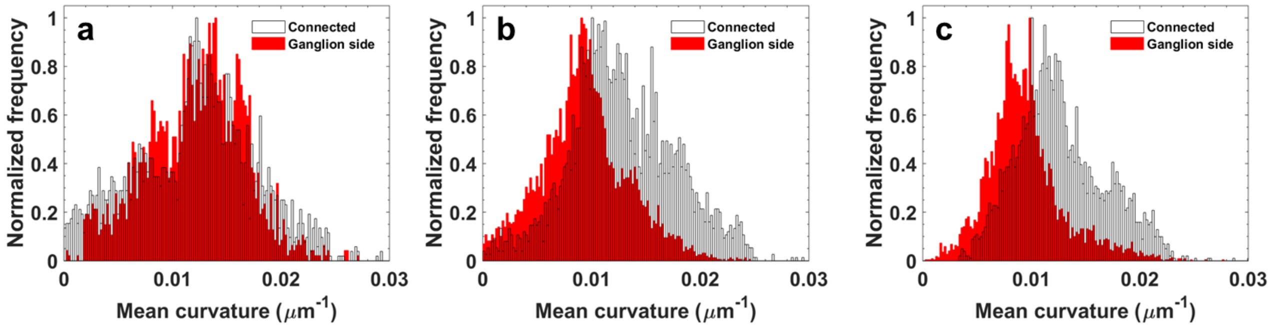

Figure S7. Comparison of the distribution of the mean curvature of brine-oil interfaces on either side of a snapping-off throat. (a) t = 111 min 28 s. (b) t = 118 min 26 s. (c) t = 119 min 4 s.

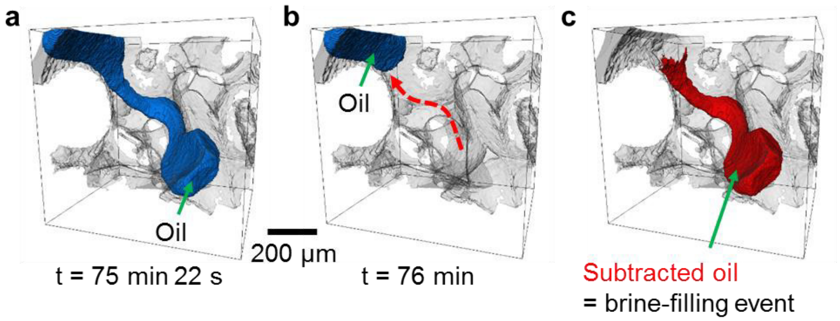

Figure S8. Pore-filling events. (a) & (b) Oil (blue) at t = 75 min 22 s and t = 76 min respectively. The dashed-red arrow shows the direction of interface migration between consecutive images. (c) Subtracted oil from consecutive images (a & b), the volume of which represent the size of a brine-filling event. Here, the brine is transparent and rock semi-transparent for effective visualization.
